# Post-Traumatic Stress Disorder in ICU Survivors: Correlations with Long-Term Psychiatric and Physical Outcomes

**DOI:** 10.3390/ijerph22030405

**Published:** 2025-03-10

**Authors:** Valerio Dell’Oste, Maria Martelli, Sara Fantasia, Debora Andreoli, Berenice Rimoldi, Andrea Bordacchini, Silvia Pini, Claudia Carmassi

**Affiliations:** 1 Department of Clinical and Experimental Medicine, University of Pisa, 56126 Pisa, Italy; valerio.delloste@gmail.com (V.D.); dr.fantasiasara@gmail.com (S.F.); d.andreoli1@studenti.unipi.it (D.A.); b.rimoldi@studenti.unipi.it (B.R.); a.bordacchini@studenti.unipi.it (A.B.); 2 Department of Mental Health and Addiction, Azienda USL Toscana Nord-Ovest, 55100 Lucca, Italy; 3 Anaesthesia and Intensive Care Unit, Azienda Ospedaliero-Universitaria Pisana, 56124 Pisa, Italy; m.martelli78@gmail.com (M.M.); sipini@gmail.com (S.P.)

**Keywords:** PTSD, ICU, anxiety, depression, disability

## Abstract

Intensive care unit (ICU) admission can represent a relevant physical and psychological burden in patients, leading to long-term mental health problems such as anxiety, depression, and post-traumatic stress disorder (PTSD). The present study aimed to systematically assess the physical and psychiatric (particularly depressive, anxiety, and post-traumatic stress) symptoms in patients discharged from the ICU of a major University Hospital in Italy (Pisa) 6 months earlier, with particular, attention to differences between patients who developed PTSD and those who did not. The strength of this study is to increase the understanding of PTSD, depressive and anxiety symptoms; in particular, their correlations with the physical sequalae. Subjects were assessed six months after ICU discharge by means of the Glasgow Outcome Scale-Extended (GOS-E), Quality of Life after Brain Injury (QOLIBRI), the 3-level version of the EQ-5D (EQ-5D-3L) questionnaire, Impact of Event Scale-Revised 22-item (IES-R), Patient Health Questionnaire, 9-Item Version (PHQ-9), and Generalized Anxiety Disorder Assessment, 7-item version (GAD-7). The results of this study showed, in accordance with the IES-R, a moderate prevalence of PTSD (25.3%) six month after ICU discharge and a statistically significant higher prevalence (63.6%, *p* = 0.039) of moderate and severe disabilities in the PTSD group compared to the no-PTSD group, as well as higher depressive and anxiety symptoms and other psychiatric sequelae, suggesting the need for accurate long-term psychiatric assessment in ICU survivors.

## 1. Introduction

Intensive care unit (ICU) admission can be a relevant physical and psychological stressor as patients often face life-threatening conditions, and they can undergo sedation and invasive measures that may lead to a significant reduction in their autonomy, causing them to feel threatened and helpless in their condition [1]. This challenging situation has been shown to lead to long-term mental health problems such as anxiety, depression, and post-traumatic stress disorder (PTSD) [2,3]. The months following discharge can be crucial as patients struggle with the remission process: up to one-third of them can experience anxiety and major depression during the first year, and up to 22% can develop PTSD [4,5].

Risk factors for PTSD after ICU discharge can include variables already known to be vulnerability factors after traumatic experiences, such as female gender, younger age, pre-existing psychiatric diagnosis (e.g., anxiety disorders or major depression), low educational level, low emotional or psychosocial support after trauma; economic burden related to the event, and eventual significant losses that occurred in the framework of the same trauma [6,7,8,9,10]. Other variables can be directly attributed to the ICU experience, such as near death experiences, invasive measures, the use of sedative agents, confused memories of ICU stay, delusional experiences, and the onset of intrusive memories and nightmares during hospitalization [11,12,13]. Notably, high levels of anxiety during hospitalization are a risk factor for the onset of PTSD [6]. Studies have shown that peritraumatic stress, measured by means of the impact of event scale-revisited, just after ICU discharge can facilitate the development of PTSD, particularly in patients with a history of anxiety disorders [7].

PTSD symptoms have been detected in ICU-discharged patients. High levels of intrusive thoughts and avoidance behaviors at 3 and 9 months after discharge have been reported in patients exhibiting anxiety and depressive symptoms [14,15], and PTSD symptoms are comprised within the expression of post intensive care syndrome (PICS) [16]. Interestingly, these symptoms have sometimes been reported as more invalidating than physical symptoms and can worsen the quality of life in patients discharged from ICUs [17].

Patients admitted for acute respiratory distress syndrome (ARDS), as well as other patients needing mechanical ventilation, are particularly at risk of developing neuropsychiatric consequences: rates up to 36% of depressive symptoms and up to 62% of anxiety symptoms have been reported, while a third of subjects can develop PTSD within the first year from discharge [18]. In the last 20 years, coronavirus pandemics such as severe acute respiratory syndrome (SARS) and Middle East respiratory syndrome (MERS), gave us the opportunity to verify the notable impact of PTSD, depression, and anxiety symptoms in patient admitted in ICUs for respiratory infections [19,20]. Moreover, in the recent years, the COVID-19 pandemic led to a dramatic increase in patient needing intensive care treatment, with rates up to 21% of the subjects affected experiencing acute respiratory distress [21,22], and a number of studies highlighted the relevant rates of psychiatric symptoms in COVID-19 survivors [23,24,25,26], including healthcare workers [27,28].

The severity of PTSD symptoms has been related to disability levels, as previous studies showed how patients with multiple diagnoses, commonly polytraumatic injuries, tend to have the worst course of recovery [29,30]. This can be a relevant factor as we know that a substantial percentage of COVID-19 patients can develop long-lasting and debilitating consequences of the infection, known as Long COVID [31], which symptoms can overlap with PICS in ICU survivors [32], worsening the quality of life and mental health outcomes.

To the best of our knowledge, there is little data in the literature on physical and psychiatric sequelae after ICU discharge, particularly in Italy and with follow-up in the same hospital where the patient had been admitted to the ICU. Given the relevance of PTSD after ICU discharge, the aim of the present study was to systematically assess PTSD 6 months after discharge from ICU of a major university hospital in Italy for a severe medical life-threatening condition. Assessments included the evaluation of the presence of anxiety and depressive symptoms, as well as for the physical sequelae. The secondary aim of the present study was thus to compare these variables between patients who developed PTSD and those who did not. It is also interesting to note that the cause of the ICU hospitalization of some patients in our study was COVID-19 infection, in which Italy was the first affected country in Europe where the same ICUs had to simultaneously admit patients who required hospitalization for the worsening severity of the COVID-19 infections and others requiring admission for traumatic or medical illness not related to COVID-19 [33]. In addition, for the first time, our study combines anesthesiologic and psychiatric evaluations, simultaneously analyzing physical and psychological impact and the correlations between the two.

## 2. Materials and Methods

### 2.1. Study Sample and Procedures

In the present study we systematically recruited patients discharged from the ICU of the Azienda Ospedaliero Universitaria Pisana (AOUP, Pisa, Italy) in the period between March 2021 and November 2021, for a six-month follow-up after discharge. Every patient accepted to take part to the study at the time of discharge. All enrolled patients were evaluated at a 6-month follow-up visit (September 2021 to May 2022). Patients enrolled in the study were admitted to the ICU for different reasons, such as COVID-19-related severe respiratory distress, peritonitis, mesenteric ischemia, acute abdomen, BPCO exacerbation, acute pancreatitis, septic shock, poly-trauma, respiratory failure, myocardial infarction, and postoperative pancreatic cancer. The inclusion criteria were a minimum of 5 days of ICU hospitalization and being older than 18 years. Exclusion criteria included admission in the ICU due to traumatic brain injury, a lack of collaboration skills, and/or verbal communication impairments that would have interfered with the patient’s ability to follow the protocol’s assessments (e.g., fluency in Italian). Experienced and trained anesthesiologists employed in the ICU, as well as psychiatrists and residents in psychiatry from the psychiatric clinic of the same AOUP, University of Pisa, Pisa, Italy, performed the assessments. For the physical assessment of quality of life and overall disability, the following metrics were used: the Glasgow Outcome Scale-Extended (GOS-E), a structured interview designed to assess the general level of disability and recovery from traumatic brain injury [34,35]; the three-level EQ-5D version (EQ-5D-3L), a set of measures used to assess and characterize overall health (EQ-5D, online website); the Quality of Life after Brain Injury (QOLIBRI), which is a survey instrument designed to assess the health-related quality of life (HRQoL); and the impact of rehabilitation or other interventions after traumatic brain injury [36]. For psychiatry assessments, the following were used: the Event Scale-Revised 22-item (IES-R), which is an assessment tool for the PTSS [37,38]; the PHQ-9, a questionnaire that indicates the existence of depressed symptoms [39,40]; and the GAD-7, an instrument for identifying anxiety symptoms [41]. After being fully informed about the study and given the opportunity to ask questions, patients gave their informed consent to participate. The study was carried out in compliance with the Declaration of Helsinki and with the approval from the Ethics Committee of the Area Vasta Nord-Ovest Toscana (Italy, protocol number 17152/2020). In addition, confidentiality and data protection were maintained during the study, such as using codes instead of participants’ real names and secure data storage.

### 2.2. Assessment Instruments

The GOS-E is an evaluation of overall disability and outcome after a traumatic brain injury, and it is a clinician-administered evaluation method [34,35]. In order to improve the limitations of the first GOS scale, which was created in 1975 [42], a new scale known as the GOS-E was created [43]. To further stratify the lower and upper levels of functioning assessed by the GOS scale, the GOS-E has been developed as an 8-point scale [43]: upper good recovery, lower good recovery, upper moderate disability, lower moderate disability, upper severe disability, lower severe disability, vegetative state, and dead [35,44]. In order to simplify the measurements, in the present study, the GOS-E scale was divided into two levels of functioning: absence of disability and moderate/severe disability. In our study, this evaluation was performed by skilled ICU residents.

The EuroQol group developed the EQ-5D-3L scale in 1990 [45]. It is a self-report scale used to assess general health and is divided into two sections: the EQ visual analog scale (EQ VAS) and the EQ-5D-3L descriptive system. In the present investigation, we decided to use the EQ-5D-3L descriptive system, which consists of five dimensions: anxiety/depression, pain/discomfort, self-care, mobility, and habitual activities [46]. Three functioning levels are assigned to each dimension: extreme problems, some problems, and no problems. We split the sample into two groups in order to simplify the results: the presence of problems (some problems plus extreme problems) and the absence of problems.

The QOLIBRI is aimed at determining the HRQoL of patients following a traumatic brain injury and the effects of rehabilitation or other treatments [36]. It is structured as a 37-item questionnaire focusing on six aspects of HRQoL after a brain injury, and it is a self-report questionnaire [36]. It is also divided into two sections: Section A (level of satisfaction) and Section B (domain of complaints), and it provides a quality-of-life profile as well as a total score. Together, the two sections make up six scales: ‘Physical problems’ (5 items), ‘Emotions’ (5 items), ‘Social relationships’ (6 items), ‘Self’ (7 items), ‘Cognition’ (7 items), and ‘Daily life and autonomy’ (7 items) [36]. A five-point rating scale is used to rate each item: 1 (not at all), 2 (slightly), 3 (moderately), 4 (quite), and 5 (very) [36]. The total score that the QOLIBRI scale produces is the total of the scores that are obtained in each of the subscales [36]. In the present investigation, we decided to use the QOLIBRI total scale, which was recently verified in relation to the QOLIBRI subscales, and it comprises six items: ‘Physical problems’, ‘Emotions’, ‘Social relationships’, ‘Self’, ‘Cognition’, and ‘Daily living and autonomy’ [47]. A five-point rating scale is used to rate each of the six items: 1 (not at all), 2 (slightly), 3 (moderately), 4 (quite), and 5 (very) [47]. After adding the scores for all the items, the overall score is transformed into a percentage scale: the quality-of-life ranges from 0 (the lowest possible) to 100 (the highest possible) [47]. The QOLIBRI scales demonstrate strong internal consistency and test–retest reliability, meeting standard psychometric criteria [48,49]. The GOS-E and QOLIBRI questionnaires are frequently used in different therapeutic settings, although they were specifically designed for people with brain injury.

PTSD was assessed 6 months after the discharge from ICU by means of the IES-R [37,38], a self-report questionnaire with good internal consistency [50]. The IES-R questionnaire is frequently used to assess trauma and stress-related symptoms in rescue workers [51,52,53]. It uses a 5-point rating scale to score each item. A total score of 24 or more indicates moderate to severe post-traumatic stress symptoms and is suggestive of PTSD [50,54]. When filling out the questionnaire, patients were asked to refer to the traumatic event during admission to the ICU due to a life-threatening illness.

Depressive symptoms were assessed by means of the PHQ-9. This self-report questionnaire comprises nine self-related items on a scale from 0 (not at all) to 3 (almost every day), determining an available range of 0–29 with possible scores of 0–4, 5–9, 10–14, 15–19, and 20–27 that indicate minimal, mild, moderate, moderately severe, and severe depressive symptoms, respectively [39,40,55]. The PHQ-9 questionnaire has demonstrated high agreement with the major depression diagnosis made with structured interviews [56,57,58].

Anxiety symptoms were assessed by means of the GAD-7 questionnaire, which has been demonstrated to be valid and accurate with strong internal consistency and test–retest reliability [41]. It is a self-report scale that consists of 7 items and is frequently used to assess generalized anxiety disorders. The patient is asked to estimate how often he/she has experienced the seven typical symptoms of anxiety disorders over the previous two weeks, indicating a value from 0 to 3 for each item. Minimal, mild, moderate, and severe anxiety symptoms are represented by scores of 0–4, 5–9, 10–14, and 15–21, respectively [41,59].

### 2.3. Statistical Analysis

All statistical analyses were performed using the Statistical Package for Social Science (SPSS), version 26.0. Continuous variables were expressed as the mean ± standard deviation (SD), whereas categorical variables were reported as percentages. All tests were two-sided, and a *p* value < 0.05 was considered statistically significant. Chi-square (or Fisher when appropriate) was calculated to assess differences between subjects with PTSD and no PTSD ones. To compare continuous normally distributed variables (age and QOLIBRI scores) among groups, the *t*-test was used, while the Man-Whitney test was used for the non-parametric ones (length of hospitalization, GAD-7, and PHQ-9 scores).

## 3. Results

### 3.1. Socio-Demographic Characteristics

A total sample of 143 patients discharged from the ICU of the AOUP satisfied the inclusion criteria of the present study. Among these patients, five had died, twenty refused to participate in the study, and twenty-seven could not be contacted because of reallocation or a change in contact data. The final sample included 87 participants (N = 65, 74.7% males and N = 22, 25.3% females). In accordance with the IES score, the total sample was divided into two groups: 22 patients (25.3%) in the PTSD group and 65 patients (74.7%) in the no-PTSD group. The mean age of the whole sample was 53.73 ± 17.44 years, with a statistically significant difference between the PTSD and no-PTSD groups (47.81 ± 17.07 and 55.73 ± 15.67 years, respectively, *p* = 0.048), as shown in Table 1.

### 3.2. Clinical Characteristics

The GOS-E simplified test showed a statistically significant higher prevalence of moderate and severe disabilities in the PTSD group (63.6%) compared to the no-PTSD group (35.4%) (*p* = 0.039). The PTSD group presented the lowest quality of life when referring to the item of emotions in the QOLIBRI scales compared to the no-PTSD group (10.55 ± 4.48 vs. 13.03 ± 3.95, *p* = 0.016). Furthermore, the PTSD group reported more problems in the anxiety/depression dimension of the EQ-5D-3L scale (59.1% vs. 26.2%, *p* = 0.11). Confirming these data, the PTSD group experienced more severe anxiety symptoms, as evaluated with the GAD-7 questionnaire (8.09 ± 5.85 vs. 2.58 ± 4.03, *p* < 0.001), and more severe depression symptoms, as evaluated with the PHQ-9 questionnaire (9.63 ± 4.73 vs. 2.95 ± 3.15, *p* < 0.001), compared to the no-PTSD group (see Table 1).

**Figure 1:**
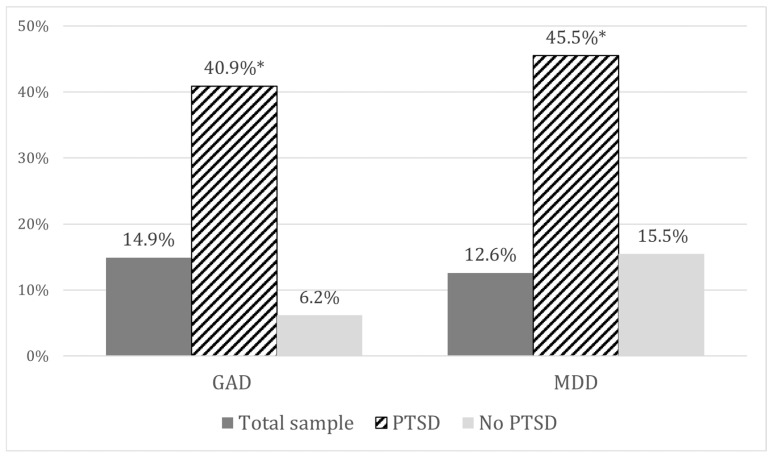
Rates of GAD and MDD in the total sample (N = 87) and in the PTSD (N = 22) and no-PTSD (N = 65) groups; *p* < 0.001. “*” is used to highlight statistically significant results.

When comparing the rates, the anxiety symptoms were reported in 40.9% of PTSD patients, with statistically significant differences (*p* < 0.001) compared to the total sample (14.9%) and to the no-PTSD group (6.2%). Referring to the rates of depressive symptoms, there were statistically significant differences (*p* < 0.001) between the PTSD group (45.5%), the total sample (12.6%), and the no-PTSD group (15.5%), as shown in Figure 1.

## 4. Discussion

The present study showed moderate rates of PTSD in a sample of patients that had been hospitalized 6 months earlier in the ICU of a major university hospital, with about one-fourth of the total sample being affected. Our results are in line with previous studies reporting PTSD rates ranging from 21% to 35% [4,5]. Furthermore, according to the literature, younger age seemed to be associated with a high frequency of PTSD development after ICU discharge. Interestingly, neither the type of diagnosis for ICU admission nor the duration of hospitalization significantly affected the development of PTSD. This suggests no significantly higher risk to develop PTSD in patients admitted fot COVID-19 infection with respect to other causes unlike some previews reports [23,24,25,26]. Studies have suggested a major role of the extent of the acute trauma that the patient suffered before and during hospitalization, and this may be corroborated by our results [6,7,8,60] showing a statistically significant higher prevalence of moderate and severe disabilities in the PTSD group compared to the no-PTSD group. This is also in line with previous evidence reporting an association between the incidence of PTSD and the years of life lived with disability in survivors [60].

Regarding other psychiatric sequelae, our results showed a lower quality of life when referring to the item of emotions (loneliness, boredom, anxiety, depression, and anger/aggression) in the QOLIBRI scale in the PTSD group compared to the no-PTSD group. Some authors suggested a major role of these symptoms, even more than physical symptoms, in worsening the quality of life in patients discharged from ICUs [17]. These results are also confirmed by our results on the GAD-7 and PHQ-9 scale scores, where the PTSD group experienced more severe anxiety and depression symptoms compared to the no-PTSD group, in line with previous studies reported significant symptoms of anxiety and depression in ICU survivors in the first year after discharge [60].

Some important limitations of this study should be kept in mind when interpreting our results. The first one is the limited sample size that may have contributed to the lack of significance in some of the results of this study, which may impact the generalizability of our results. The second limitation is the lack of assessment of psychopathological symptoms before admission to the ICU, including PTSD symptoms, although we excluded from this study all patients with a previous diagnosis of psychiatric illness, as we may argue that the correlations between anxiety and depressive symptoms and PTSD may be related to a pre-existing anxiety depressive or previous post-traumatic symptomatology. We may recall that this limitation in the inward assessment was related to the organization of the clinical departments that do not include psychiatrist in the ICU ward apart from specific cases where consultation psychiatry is required. Consistently, participants were also not asked about their trauma history; this may have an impact on the results as multiple lifetime traumas may be related to an increased risk for PTSD [61,62,63]. Third, all the assessment of psychiatric symptoms are from a self-report questionnaire, which could be considered less accurate than a clinical assessment. However, there is often a strong correlation between clinical ratings and self-report results, since we only used validated scales. Moreover, another limitation of our study is the lack of a control group, limiting the possibility to determine how ICU-specific factors contribute to the observed outcomes. Other limitations can be represented by the exclusion criteria that can represent a selection bias, thereby obtaining results that are not representative of the entire population of interest, and the study’s single-center design limits the generalizability of the conclusions to a broader population. Data on the detailed description of the procedures adopted in the ICU (e.g., sedation, ventilation, or other treatment protocols) that could impact psychiatric or physical outcomes were recorded Another limitation of this study is the lack of assessment on possible emotional or psychosocial and economical support that occurred in the aftermath of discharge. By taking the aforementioned limitations into account, we acknowledge the challenges and constraints of carrying out research in an emergency setting; we believe that this unique context adds valuable insights to the literature.

## 5. Conclusions

The results of this study showed a moderate prevalence of PTSD (25.3%) in ICU survivors 6 months after discharge and a statistically significant higher prevalence of physical and psychiatric sequelae in the PTSD group compared to the no-PTSD group, suggesting the need for an accurate psychiatric assessment in this population and for targeting younger patients and those with traumatic distress in order to plan effective supportive care, tailored interventions, or long-term follow-up strategies. Our study highlights the importance of integrating early PTSD screening and mental health support into post-ICU care pathways. It would also be beneficial if future studies explored the role of ICU treatments in PTSD development and if they conducted multicenter studies to validate findings in larger populations. Additionally, further research should also examine how specific ICU care procedures may influence post-traumatic symptoms, allowing for a more precise identification of at-risk populations.

## Figures and Tables

**Table 1:** Socio-demographic and clinical characteristics in the total sample (N = 87) and in the PTSD (N = 22) and no-PTSD (N = 65) groups.

		Total Sample N = 87	PTSD N = 22	No PTSD N = 65	*p*
**Gender (Female)**	N (%)	22 (25.3)	7 (31.8)	15 (23.1)	0.595
**Age (years)**	Mean ± SD	53.73 ± 17.44	47.81 ± 17.07	55.73 ± 15.67	**0.048**
Diagnosis at ICU admission					
*Major trauma*	N (%)	42 (48.3)	15 (68.2)	27 (41.5)	-
*COVID-related acute respiratory failure*	N (%)	30 (34.5)	5 (22.7)	25 (38.5)	0.130 *
*Other medical diagnoses*	N (%)	6 (6.9)	2 (9.1)	4 (6.2)	0.732 *
*Other chirurgical diagnoses*	N (%)	9 (10.3)	0 (0.0)	9 (13.8)	0.083 *
Length of hospitalization (days)	Mean ± SD	17.76 ±15.33	18.73 ± 20.56	17.43 ± 13.29	0.601
GOS-E					
***Absence of disability***	N (%)	50 (57.5)	8 (36.4)	42 (64.6)	**0.039**
*Moderate/severe disability*	N (%)	37 (42.5)	14 (63.6)	23 (35.4)	
QOLIBRI					
*physical problems*	Mean ± SD	11.99 ± 4.65	11.09 ± 4.54	12.31 ± 4.68	0.295
*cognition*	Mean ± SD	12.65 ± 4.19	11.36 ± 4.92	13.11 ± 3.84	0.093
***emotions***	Mean ± SD	12.38 ± 4.21	10.55 ± 4.48	13.03 ± 3.95	**0.016**
*daily life and autonomy*	Mean ± SD	11.81 ± 4.46	10.91 ± 4.59	12.13 ± 4.41	0.273
*social relationships*	Mean ± SD	11.83 ± 4.57	10.50 ± 4.58	12.31 ± 4.51	0.112
*infuturation*	Mean ± SD	12.55 ± 3.83	11.36 ± 4.77	12.97 ± 3.38	0.091
*total score*	Mean ± SD	74.25 ± 21.16	67.14 ± 24.21	76.77 ± 19.55	0.066
EQ-5D-3L					
*Mobility (disturbed)*	N (%)	35 (40.2)	12 (54.5)	23 (35.4)	0.183
*Selfcare (disturbed)*	N (%)	15 (17.2)	5 (22.7)	10 (15.4)	0.516
*Usual activities (disturbed)*	N (%)	37 (42.5)	13 (59.1)	24 (36.9)	0.117
*Pain (disturbed)*	N (%)	50 (57.5)	16 (72.7)	34 (52.3)	0.154
***EQ Depression (disturbed)***	N (%)	30 (34.5)	13 (59.1)	17 (26.2)	**0.011**
**GAD-7**	Mean ± SD	3.98 ± 5.13	8.09 ± 5.85	2.58 ± 4.03	**<0.001**
**PHQ-9**	Mean ± SD	4.64 ± 4.62	9.63 ± 4.73	2.95 ± 3.15	**<0.001**

* With respect to major trauma. Statistically significant parameters and respective *p*-value are highlighted in bold

## Data Availability

The data that support the findings of this study are available from the corresponding author upon reasonable request.
